# An overview of current COVID-19 clinical trials and ethical considerations editorial

**DOI:** 10.1016/j.amsu.2020.08.041

**Published:** 2020-08-29

**Authors:** Brad Boserup, Mark McKenney, Adel Elkbuli

**Affiliations:** Department of Surgery, Division of Trauma and Surgical Critical Care, Kendall Regional Medical Center, Miami, FL, USA; Department of Surgery, Division of Trauma and Surgical Critical Care, Kendall Regional Medical Center, Miami, FL, USA; University of South Florida, Tampa, FL, USA; Department of Surgery, Division of Trauma and Surgical Critical Care, Kendall Regional Medical Center, Miami, FL, USA

**Keywords:** Evidence-based medicine, Clinical trials, Ethical considerations, COVID-19 pandemic, Patient safety

## Abstract

Not applicable.

## Highlights

• There are currently 1676 clinical trials being conducted worldwide to assess various COVID-19 treatment modalities.
• Newer agents such as remdesivir appear to provide clinical benefit, however, despite many ongoing clinical trials, there is still a lack of definitive evidence to guide clinical decision making.
• Given the rapidly evolving COVID-19 clinical trial landscape, it is vital that the US drug-evaluation process is condcuted safely, effectively, and with high credibility.

**Fig. 1:**
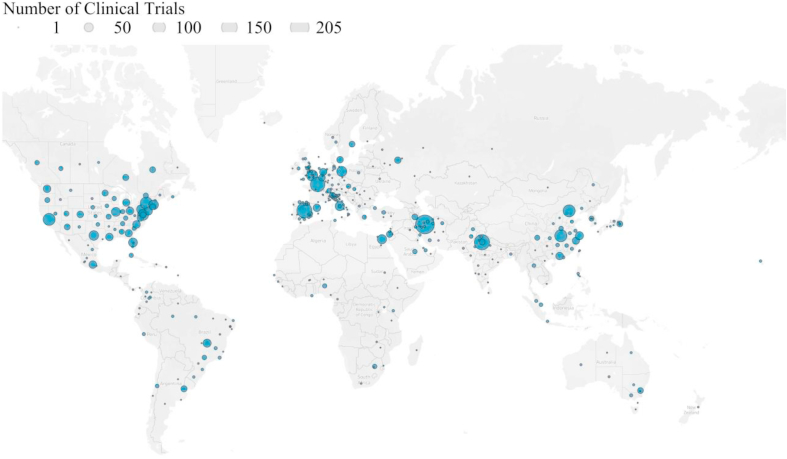
World map showing the number of clinical trials by state or province. Data obtained from the Global COVID-19 Clinical Trial Tracker as of August 25, 2020.

**Fig. 2:**
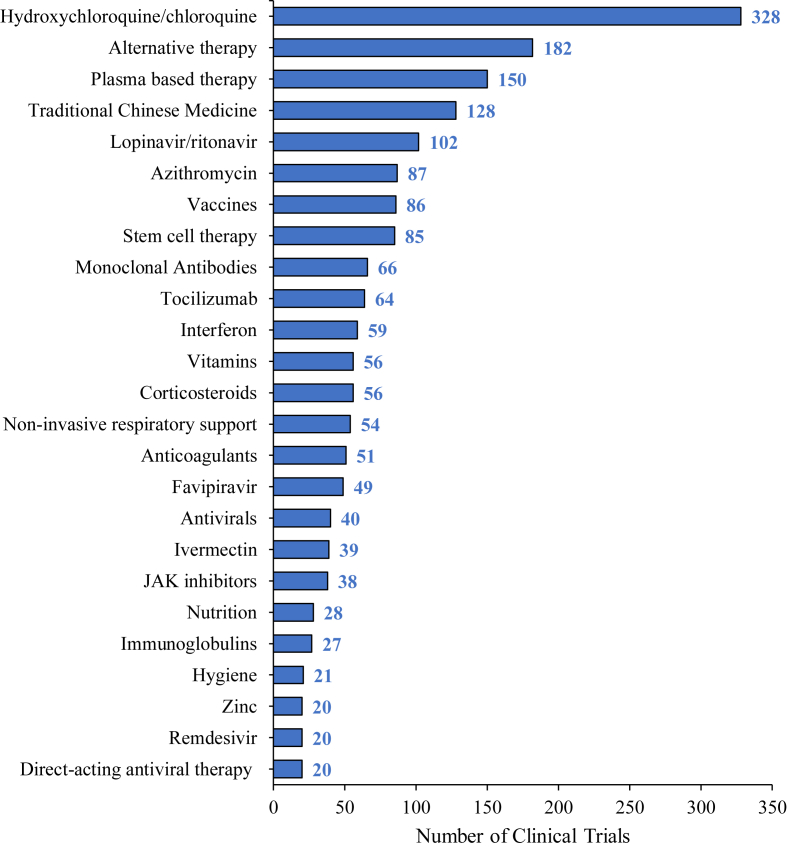
The top 25 most commonly investigated treatments according to current clinical trial numbers. Data obtained from the Global COVID-19 Clinical Trial Tracker as of August 25, 2020.

The causative agent of the COVID-19 pandemic, severe acute respiratory syndrome coronavirus 2 (SARS-CoV-2), is a highly contagious RNA virus that has spread rapidly since the initial outbreak in December 2019. By August 25, 2020, there were 5,715,567 cases and 176,617 deaths in the United States (US) alone in the absence of any Food and Drug Administration (FDA) approved drugs to treat COVID-19 [1]. However, as of August 25, 2020 there were 2103 clinical trials reported from numerous national and international trial registry sites spanning 96 countries (Fig. 1) [2]. Some of the treatment options under investigation with the greatest number of trials at the time of writing included hydroxychloroquine or chloroquine, alternative therapies, plasma-based therapy, traditional Chinese medicines and lopinavir-ritonavir (Fig. 2).

Chloroquine and hydroxychloroquine are known to have effects against several RNA viruses such as Zika and chikungunya and have exhibited an ability to inhibit SARS-CoV-2 in vitro [3,4]. Additionally, the anti-inflammatory properties of chloroquine and hydroxychloroquine may control the effects of cytokine storm seen during late stages of COVID-19 and mitigate associated tissue damage [5]. The results of one open-label non-randomized trial in France, involving 36 COVID-19 patients, found that hydroxychloroquine use was associated with significantly improved outcomes compared to controls [6]. The study found that 70% of patients treated with hydroxychloroquine had negative PCR nasopharyngeal samples at day 6 compared with 12.5% in the control group [6]. However, despite promising results, this study has since received criticisms regarding its methodology. A randomized clinical trial in Wuhan, China that included 62 patients with COVID-19, found that the use of hydroxychloroquine was associated with significantly reduced time to clinical recovery, cough remission time, body temperature recovery time, and reduced progression to severe disease (this study has yet to undergo peer review) [7]. In contrast, an open-label randomized trial in China that included 30 patients, did not find a significant difference at day 7 in viral clearance between the hydroxychloroquine treatment group and the control group [8]. Therefore, despite a large number of clinical trials underway to evaluate the efficacy of hydroxychloroquine or chloroquine in the treatment of COVID-19, there is currently an insufficient amount of published data to advocate for or against use.

Lopinavir-ritonavir is a combination protease inhibitor originally used for HIV treatment and prophylaxis, which has in vitro activity against SARS-CoV [9]. However, the clinical trial data available examining the use of lopinavir-ritonavir in the treatment of COVID-19 has shown it has little to no benefit in the treatment of COVID-19. One open-label randomized controlled trial in China, with 199 patients with COVID-19, found that the use of lopinavir-ritonavir was not associated with any difference in time to clinical improvement compared to standard care [10]. These results are understandable since lopinavir-ritonavir was originally designed to fit the HIV protease C2-symmetric binding pocket which is not present in SARS-CoV-2 proteases [11]. Additionally, the nucleoside analog favipiravir has recently been shown to be more effective than lopinavir-ritonavir. An open-label non-randomized comparative control trial in China that included 80 patients found that use of favipiravir was associated with significantly shorter viral clearance times, and significantly improved chest imaging compared to the use of lopinavir-ritonavir [12]. The improved efficacy of favipiravir versus lopinavir-ritonavir is likely due to its ability to act as a chain terminator and inhibit viral RNA polymerase [13].

Similarly, the nucleotide analog remdesivir, which also inhibits viral RNA polymerase has recently garnered a great deal of interest following the release of preliminary data from the Adaptive COVID-19 Treatment Trial [14]. This randomized, double-blind, placebo-controlled trial that included 1059 patients with COVID-19 found that the use of remdesivir was associated with faster recovery times (11 days versus 15 days with placebo) and reduced 14-day mortality rates (7.1% versus 11.9% with placebo) [15]. Lastly, some other potentially useful treatment modalities include the use of convalescent plasma therapy and IL-6 inhibitors. Thus, despite a large number of ongoing clinical trials, the results from most are still pending, and there is currently still a paucity of quality information to guide clinical decision making. However, hundreds of trials are currently recruiting participants to investigate other potential treatment options, and more information from ongoing trials should become available in the coming months.

Amidst the fervor surrounding the development of new COVID-19 treatments, it is paramount that researchers maintain data integrity, especially given several recent high-profile retractions. These recent events highlight the need for further data oversite to maintain the integrity of new evidence. Furthermore, it is important that researchers also consider the ethical implications surrounding the emergency acceleration of clinical trials and approval processes. For instance, phase 3 clinical trials typically last between 1 and 4 years, and remdesivir which is currently just beginning phase 3 testing (the earliest ongoing trial was posted to ClinicalTrials.gov on February 21, 2020; Identifier: NCT04280705) received an Emergency Use Authorization (EUA) on May 1, 2020, for the treatment of hospitalized COVID-19 patients [16]. While accelerated measures may be called for, given the severity of the current pandemic, it is also crucial that this acceleration process does not jeopardize the US drug-evaluation process with regard to safety, efficacy, and credibility. Since the very nature of randomized controlled trials poses ethical dilemmas given the current climate, it is vital that all researchers and physicians abide by their continual commitments to the principles of respect for all persons, beneficence, and justice during these trying times.

## Provenance and peer review

Not commissioned, externally peer reviewed.

## Funding

None.

## Sources of funding

None.

## Ethical approval

Not applicable.

## Research registration Unique Identifying number (UIN)

1. Name of the registry:

2. Unique Identifying number or registration ID:

3. Hyperlink to the registration (must be publicly accessible).

Not applicable-no human subjects or research participants’ data were utilized or collected.

## Author contribution

Study design and conception: Adel Elkbuli, Brad Boserup.

Data collection, interpretation and analysis: Brad Boserup, Adel Elkbuli.

Manuscript preparation: Brad Boserup, Adel Elkbuli.

Critical revision of manuscript: Brad Boserup, Adel Elkbuli, Mark McKenney.

All authors read and approved the final manuscript.

## Guarantor

Mark McKenney.

## Declaration of competing interest

Authors declare no competing interests.
